# Novel Noxipoint Therapy versus Conventional Physical Therapy for Chronic Neck and Shoulder Pain: Multicentre Randomised Controlled Trials

**DOI:** 10.1038/srep16342

**Published:** 2015-11-10

**Authors:** Charles C. Koo, Ray S. Lin, Tyng-Guey Wang, Jau-Yih Tsauo, Pan-Chyr Yang, Chen-Tung Yen, Sandip Biswal

**Affiliations:** 1 Stanford University, Stanford, CA 94305, U.S.A.; 2 College of Medicine, National Taiwan University, Taipei, Taiwan 100; 3 Pain Cure Centre, Palo Alto, CA 94306, U.S.A.; 4 Department of Physical Medicine & Rehabilitation, National Taiwan University Hospital, Taipei, Taiwan 100; 5 School and Graduate Institute of Physical Therapy, National Taiwan University, Taipei, Taiwan 100; 6 Institute of Biomedical Sciences, Academia Sinica, Taipei, Taiwan 115; 7 Department of Life Science, National Taiwan University, Taipei, Taiwan 106; 8 Diagnostic Radiology, Department of Radiology, Stanford University Medical Centre, Stanford, CA 94305, U.S.A.

## Abstract

As chronic pain affects 115 million people and costs $600B annually in the US alone, effective noninvasive nonpharmacological remedies are desirable. The purpose of this study was to determine the efficacy and the generalisability of Noxipoint therapy (NT), a novel electrotherapy characterised by site-specific stimulation, intensity-and-submodality-specific settings and a immobilization period, for chronic neck and shoulder pain. Ninety-seven heavily pretreated severe chronic neck/shoulder pain patients were recruited; 34 and 44 patients were randomly allocated to different treatment arms in two patient-and-assessor-blinded, randomised controlled studies. The participants received NT or conventional physical therapy including transcutaneous electrical nerve stimulation (PT-TENS) for three to six 90-minute sessions. In Study One, NT improved chronic pain (−89.6%, Brief Pain Inventory, p < 0.0001, 95% confidence interval), function (+77.4%, range of motion) and quality of life (+88.1%) at follow-up (from 4 weeks to 5 months), whereas PT-TENS resulted in no significant changes in these parameters. Study Two demonstrated similar advantages of NT over PT-TENS and the generalisability of NT. NT-like treatments in a randomised rat study showed a similar reduction in chronic hypersensitivity (−81%, p < 0.01) compared with sham treatments. NT substantially reduces chronic neck and shoulder pain, restores function, and improves quality of life in a sustained manner.

Chronic pain affects 115 million people and costs $600 billion annually in the United States alone, yet effective treatments are lacking^1^. Standard noninvasive treatments include physical therapy (PT) and analgesics. The prescription of analgesics such as opioids, which provide temporary relief from chronic pain, has increased rapidly in the past two decades, along with proportional increases in analgesic abuse and addiction. Effective noninvasive nonpharmacological remedies are highly desirable, as healthcare costs and drug abuse are mounting^2^.

Transcutaneous electrical nerve stimulation (TENS) is a commonly used PT treatment modality for pain. Despite the long history of the use of TENS for the treatment of chronic pain conditions, its effectiveness has been inconclusive based on prior randomised controlled trials (RCTs). Multiple studies showed the benefits of TENS^3^,^4^,^5^,^6^,^7^,^8^,^9^,^10^,^11^,^12^,^13^,^14^,^15^,^16^, whereas others showed no significant effects of TENS^17^,^18^,^19^,^20^,^21^. The prevailing conclusion regarding the pain-reducing effect of TENS is that it is not medically significant (pain reduction of less than 2 on a 10-point scale), although it has been statistically significant (p < 0.05) in some trials^6^,^14^, particularly in the short term^22^,^23^. Additionally, the lack of appropriate standard guidelines for TENS may have led to variability in clinical outcomes of TENS.

Noxipoint™ therapy (NT), a novel therapy utilising electrical stimulation characterised by precise electrode placement (on “Noxipoints”), intensity- and submodality-specific settings (eliciting soreness/dull pain but not sharp pain) and brief stimulation, was preliminarily found to substantially and persistently restore function and relieve chronic pain in a large cohort of chronic pain patients over the past decade. Two independent RCT studies were conducted. The primary objective of Study One (in the U.S.) was to evaluate the efficacy of NT compared to conventional PT including TENS (PT-TENS, which included exercise, manual and heat therapies and TENS) for the treatment of chronic neck and shoulder pain based on relative changes in a patient-reported pain scale, functional impairment, and quality of life (QOL) indices from baseline (pre-treatment assessment) to follow-up (from 4 weeks after the last treatment session, or approximately 8 weeks after the baseline assessment). The secondary objective was to evaluate the incremental changes in the above outcomes during treatment. The primary objectives of Study Two (in Taiwan) were to verify the findings of Study One using similar outcome measures of chronic neck pain in a geographically different population and to evaluate the generalisability of NT performed by newly trained therapists.

In addition, a randomised study in rats was conducted to establish the translational basis for future research on the physiological mechanism of NT. This study examined the effect of NT-like treatment for induced chronic hyperalgesia via intramuscular acid injection^24^,^25^,^26^, a commonly used animal model of inflammatory pain that likely mimics the conditions of tissue injury such as sprains and myositis^26^.

## Results

**Figure 1:**
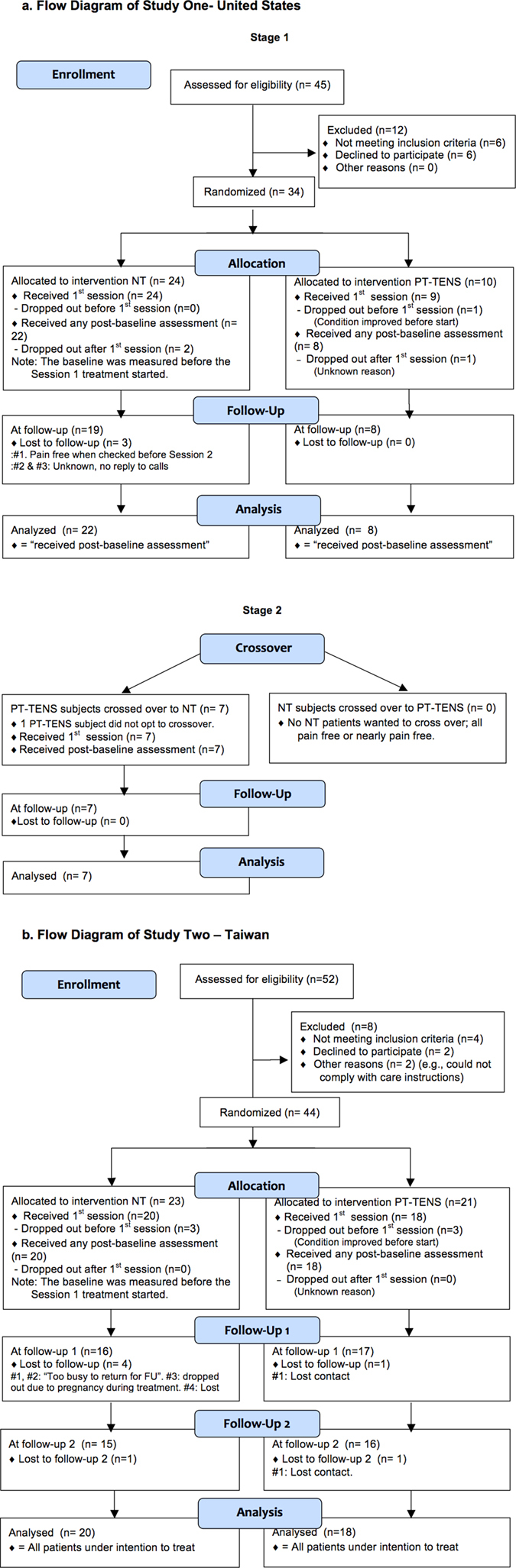
CONSORT Participant Flowchart. (**a**) Flow Diagram of Study One- United States. (**b**) Flow Diagram of Study Two—Taiwan. All randomised patients except those who did not receive at least one post-treatment assessment were included in the analysis to avoid potential bias introduced by excluding dropout patients. The missing data for the outcome measures of these dropout patients (e.g., the 4 patients lost to Follow-up 1 (FU-1) and one patient lost to Follow-up 2 (FU-2) in the NT arm of Study Two) were conservatively imputed based on the LOCF method. Thus, the number of patients analysed was greater than the number of patients observed at follow-up in both studies. The analysed Ns in Study Two were the same as the Ns of the intention-to-treat populations. In Study Two, the protocol required the collection of BPI data before each session and at each follow-up, whereas ROM data was collected only at baseline and at follow-up. As the five dropout patients did not return for either follow-up, there was no ROM measure aside from the pre-treatment measure. These data were excluded from ROM analysis, as carry-over from the pre-treatment baseline data to the missing data at FU1 and FU2 would be misleading because their corresponding BPI values showed substantial pain reduction.

Thirty-four patients were randomly allocated to different treatment arms in Study One (24 in the NT arm and 10 in the PT-TENS arm; Fig. 1). The patients in the two arms exhibited similar baseline characteristics (Table 1), and all were heavily pretreated (average pain history of 6.7 years in both arms; 2.6 and 3.0 distinct treatment classes in the NT and the PT-TENS arms, respectively). The Neck/shoulder-pain patients were distributed comparably between the NT (75%/25%) and PT-TENS arms (80%/20%). In the first stage of Study One, 30 patients receiving at least one post-baseline assessment were included in the analysis. Additionally, 44 neck-pain patients who provided written consent were randomly allocated to different treatment arms in Study Two (23 in the NT arm and 21 in the PT-TENS arm; Fig. 1), and 38 patients receiving at least one post-baseline assessment were included in the analysis (Table 1). Nearly all patients in both arms completed the assigned treatment.

None of the patients in Study One received off-protocol treatments. In Study Two, 3 of the 20 NT patients received off-protocol treatments and 13 of the18 PT-TENS patients received off-protocol treatments after the first follow-up due to on-going pain, introducing bias toward the conservative treatment in this superiority trial (see details in Supplementary Text 1).

Chronic pain, especially that of the heavily pretreated patients in Study One, was predominantly idiopathic at baseline. Based on the patient histories, seven of the 30 analysed patients in Study One reported past muscle-impairing events (e.g., car accident, sports injury, or work-related injury). Although most patients had no clear diagnosis or existing radiological evidence of injury at the beginning of this study, many patients recalled strained muscles, whiplash, pain, etc. associated with these events. Other patients reported gradual degeneration (e.g., due to bad posture at work) or a diagnosis of osteoarthritis or could not recall any specific cause of their pain. Study Two focused on chronic neck pain, excluding spondylosis with specific evidence of nerve root compression, implicating muscle or soft tissue impairment.

### Primary analysis

The primary analysis compared the outcomes between baseline and follow-up, and the results demonstrated statistically significant (p < 0.05) and clinically substantial benefits (net pain reduction on a numeric rating scale of >−2.5 or a final raw score of <3.5 for chronic pain, as defined in^27^) of NT/crossover-to-NT (XO) compared to PT-TENS with respect to pain, function, and quality of life in both studies (Tables 2 and 3).

The normality test in Study One (quantile-quantile plot) indicated that the data were marginally normal due to the relatively small sample size in the PT-TENS arm. Most variables associated with the determination of the changes from pre- to post-treatment in the NT group and the differences between the NT vs. PT-TENS arms passed the Shapiro-Wilk normality test (18 of the 20 variables) in Study Two; therefore, a paired t test was appropriate. Nevertheless, Wilcoxon’s tests, nonparametric methods that make no assumptions regarding the distribution of the variables, were conducted in addition to t tests to ensure the robustness of the analysis. All p-values from the Wilcoxon’s tests were in the same range as those from the t tests (Tables 2 and 3).

In the first stage of Study One (Table 3A), NT reduced the Brief Pain Index (BPI) “at its worst” score by 89.6% (95% confidence interval (CI), from 7.7 at baseline to 0.8 at follow-up post-NT, p < 0.0001), whereas patients in PT-TENS reported no significant change (95% CI, from 8.1 to 8.2, P = 0.84). The between-arm comparison of BPI “at its worst” revealed a significant and substantial difference (Δ = −7.0, 95% CI, −8.5 to −5.6, P < 0.0001, Table 3A). The BPI measures of “average” (Δ = −5.3, 95% CI, P < 0.0001,), “right now” (Δ = −6.0, 95% CI, P < 0.0001), and “interference with functions” (Δ= −23.4, 95% CI, P = 0.0021,), as well as functional impairment (Δ= −9.2, P = 0.002, 95% CI), were substantially and significantly different between the treatment arms (Table 2A). Regardless of the aetiology or history, NT substantially reduced or eliminated pain and restored function, and its benefits persisted to follow-up.

In the second stage, the average BPI “at its worst” in the crossover-to-NT patients was reduced by 84%, from 8.1 at the start of crossover to 1.3 (95% CI, p < 0.0001) at follow-up (an average of 5 weeks after the final treatment session). The patients who received PT-TENS maintained pain levels similar to those observed in Stage 1 after follow-up; thus, 7 of the 8 PT-TENS patients crossed over to NT. Alternatively, no patients receiving NT crossed over to PT-TENS because all NT patients reported being pain-free (74%, 14 patients, BPI = 0) or nearly pain-free (26%, 5 patients, mean BPI = 1.3 and all BPIs ≤2) at follow-up (see additional details in Supplementary Figure 1) and thus had no motivation to cross over. Note that the patients remained blinded to their treatment throughout the study.

The patients receiving NT exhibited reductions in the “average” and “right now” BPI scores by 88% and 90% (4.6- and 4.7-point reductions), respectively, and improvements in the functional impairment score by 77.4% and in QOL by 87%. All differences were highly significant (p < 0.0001, 95% CI). In contrast, PT-TENS patients showed no significant changes in all measures, except a 1.2-point reduction in BPI “right now”. After the PT-TENS patients crossed over to NT (N = 7), they experienced statistically significant improvements in all measures at magnitudes similar to those observed in the NT arm (Table 3A). The differences in the mean changes in outcome between the NT, crossover-to-NT versus PT-TENS were clinically substantial and statistically significant across all measures (Table 2A).

In Study Two, improvement in all measures was observed at the 4-week follow-up in both NT and PT-TENS patients (all statistically significant, except BPI “right now” in the PT-TENS arm, Table 2B). NT resulted in greater improvement in all measures than PT-TENS: BPI “at its worst”: improvement of 4.5 (P < 0.0001, 95% CI) versus 1.8 (P = 0.0057, 95% CI) points; BPI “average”: improvement of 3.6 (P < 0.0001, 95% CI) versus 0.9 (P = 0.064, 95% CI) points; BPI “right now”: improvement of 3.0 (P < 0.0001, 95% CI) versus 0.4 (P = 0.37, 95% CI) points; functional impairment: improvement of 8.1 (P = 0.0002, 95% CI) versus 3.6 (P = 0.02, 95% CI) points; and QOL: improvement of 27.8 (P < 0.0001, 95% CI) versus 11.0 (P = 0.0056, 95% CI) points. All differences in these improvements between the NT and PT-TENS arms were statistically significant (Table 2B). A similar trend (with slightly smaller differences between the NT and PT-TENS arms) was observed at the 8-week follow-up, although more PT-TENS patients reported receiving off-protocol treatments before the 8-week follow-up (Table 2B).

Follow-ups were conducted between 2012/03/30 and 2013/01/04 in Study One and between 2013/05/29 and 2014/04/25 in Study Two. Study One was terminated when both arms reached the target numbers of subjects established in the protocol. Study Two was terminated when the scheduled end-date of the protocol was reached near the end of the funding cycle.

### Secondary analysis

**Figure 2:**
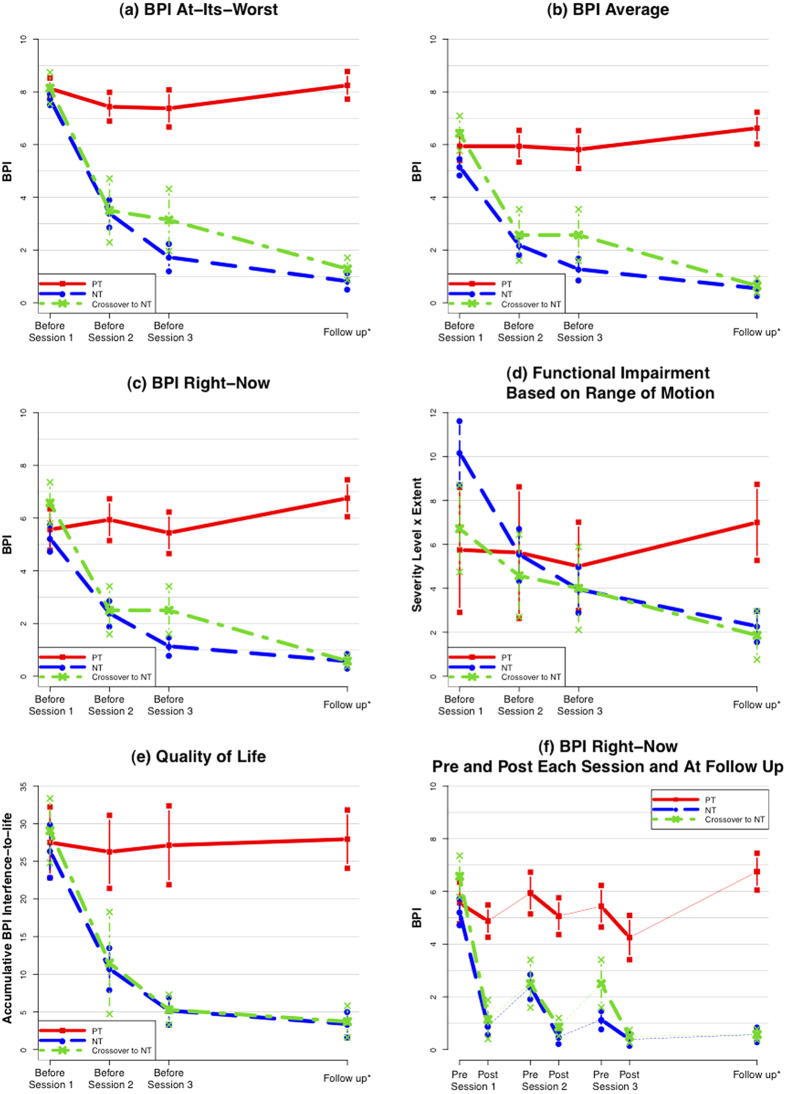
Incremental Changes in Primary and Secondary Outcomes. 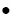
NT = Noxipoint™ therapy arm. PT = Physical therapy (including TENS) arm. Crossover-to-NT = Patients who crossed over from PT-TENS to NT after a washout period. 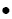
The Ns for the NT, PT, and Crossover-to-NT arms are 22, 8 and 7, respectively. 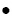
The subjects were stratified according to the primary outcome measure (BPI “at its worst”) before randomisation. 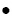
BPI “at its worst” and BPI “average” were measured based on the 48 hours preceding each session. Each session lasted 90 minutes. 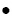
Follow-up was performed at an average of 7, 5 and 7 weeks after treatment for NT, PT, and Crossover-to-NT, respectively. 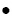
Vertical bars = standard errors.

The secondary analysis in Study One showed that NT resulted in faster improvement in all five outcome measures than PT-TENS during the treatment course (Fig. 2a–e). Patients receiving NT experienced substantial improvement in all measures after the first session and continued improvement in subsequent treatment sessions through follow-up. Figure 2f shows that all patients exhibited improved scores immediately after each session; however, the scores in the PT-TENS arm rebounded to the initial level by the subsequent session, whereas the benefit of NT was substantially retained and continued to increase over a short period (Supplementary Figures 1 and 2 and Supplementary Text 2).

### Extended follow-ups

Ten of the 26 patients in Study One who completed NT treatment (19 NT patients and 7 crossover-to-NT patients) were randomly selected for clinical follow-up after 4 to 6 months. Eight of these patients remained pain-free (80%), while the other two (20%) reported minor pain (BPI “at its worst” of 1 and 2 for each patient).

### Side effects

Some patients experienced temporary delayed-onset muscle soreness (DOMS), migrating pain, or temporary weakness of the treated area during the treatment period, as explained in the patient’s written consent form and care instructions. No significant side effects were reported.

### NT-like treatment in rats

A randomised rat study (N = 18) showed that NT-like therapy significantly (p < 0.01) and substantially reduced (by 80.7%) mechanical hyperalgesia of the hind paw induced by repeated acid injection into the gastrocnemius muscle. This reduction was observed on the second day after the therapy was administered and was sustained until the end of the study (30 days). The control group, which received sham therapy, did not exhibit significant improvement until Day 30, when spontaneous recovery occurred (see details in Supplementary Figure 3 and Supplementary Text 3).

## Discussion

The concordance of evidence across five measures in two different studies suggests that NT effectively and sustainably reduces pain, restores function, and improves quality of life and that NT is superior to conventional PT by one order of magnitude. In addition to the randomised controlled parallel study design, the crossover design of Study One efficiently reduced the influence of confounding factors because each crossover patient served as his or her own control^28^,^29^, eliminating potential assignment bias introduced by randomisation. The animal study eliminated the possibility of placebo effects, particularly as the rats in both groups were anesthetised.

Study Two was designed to evaluate the generalisability of the results from Study One in a less compliant and less heavily pretreated population with less severe conditions at baseline who were treated by newly-trained NT therapists. The patients in Study Two more frequently received off-protocol treatment and were more frequently lost-to-follow-up than those in Study One (see Supplementary Text 1). Despite these differences, NT demonstrated robust improvement and was significantly superior to PT-TENS in all outcome measures in Study Two. The difference in the percentages of patients seeking off-protocol treatment after the designated treatment between NT and PT-TENS also indicated their comparative effectiveness. Our results showed that PT-TENS provided a mild benefit to less heavily pretreated patients. This result is consistent with prior studies: among 83 published RCTs on PT modalities^3^,^4^,^5^,^6^,^7^,^8^,^9^,^10^,^11^,^12^,^13^,^14^,^17^,^18^,^19^,^20^,^21^, the greatest benefit observed in PT was a pain reduction of 14.1 in the VAS score (≈1.41 points on the BPI) after 10 treatments^14^, which is within the range of reduction in the three BPI pain subscores in the PT arm of Study Two (1.8, 0.9 and 0.4 for BPI “at its worst”, “average” and “right now”, respectively). In contrast, NT provided substantially greater pain reduction across all pain measures and even in the population in whom PT-TENS was completely ineffective.

The patient sample sizes in both studies were sufficient based on the pre-study power calculation, in which substantial effect sizes over the standard of care were selected.

NT patients experienced soreness/dull pain induced during the stimulation. 90% of them reported that the target pain was lifted after each stimulation.

Four NT patients and one PT-TENS patient (out of 33) experienced temporary soreness after TENS in Study One; the soreness typically occurred within a few hours after the electrical stimulation and disappeared within one to three days. This soreness was likely due to DOMS resulting from induced muscle contraction. Many NT patients reported that the pain “moved” from one place to another during or after each application of electrical stimulation. Such pain appeared within a few minutes, hours, or days after the initial pain disappeared. NT was applied to the sources of pain and addressed each source as it emerged. Most patients recalled that the pain from these subsequently appearing sources was pre-existing.

A few NT patients with very severe pain or limited range of motion (ROM) at baseline (<15%) reported temporary weakness in the treated area shortly after the pain had been resolved. The muscle typically regained full strength within approximately 3 to 6 days and remained pain-free. No PT-TENS patient reported a similar experience. These possible effects were explained to all patients before and during the treatment. Tissue remodelling may explain this temporary weakness and subsequent recovery.

Previously unnoticed pain often became prominent as the initial pain was reduced or eliminated in the NT group. Such “migrating pain” served as both an indicator of the recovery from the initial pain and a navigational cue indicating the next set of impaired muscles/tissues requiring treatment.

The reported BPI pain level of each patient reflected the intensity of pain at the most prominent pain sites and was independent of the number of sites treated via NT.

NT was conceived and refined over the past decade based on the treatment of over a thousand patients with various types of chronic pain, such as neck, shoulder, back, or knee pain. The key characteristics responsible for the notable advantage of NT over conventional PT, including TENS, include the following.

- It was critical to apply the stimulation to the exact location of the pair of Noxipoints™, which consistently approximated the locations of the origin and the insertion of a muscle group, coinciding with the sites containing the greatest concentration of nociceptors in the muscle^30^. Conventional TENS stimulates the general area in which the patient identifies pain. Palpation was used to locate the problem area and the Noxipoints during NT. Each area was touched briefly (for a couple of seconds) with light or moderate pressure.
- Stimulation at these paired Noxipoints must induce local soreness/dull pain (matching the characteristic sensation from C-fibres). Other induced sensations (e.g., tingling, sharp pain, or muscle contraction) in the absence soreness/dull pain did not lead to positive outcomes after stimulation in approximately 80% of cases.
- Patient compliance with the instruction to rest was critical in NT. Noncompliance almost always led to a need for repeated NT on the same muscle/tissue. This phenomenon suggests a role of cell remodelling/regeneration in the effects of NT. The estimation of the resting period described in the Intervention section was based on the observed duration of skeletomuscular cell repair mediated by myosatellite cells in the laboratory^31^,^32^ and on the empirical observation of NT in preliminary studies. Satellite cells (unipotent stem cells, or more precisely, progenitor cells) are committed to the late stage of the myotube differentiation and, thus, differentiate into muscle cells^33^ within a few days. In contrast, in conventional PT or TENS, the patient is encouraged to actively exercise the treated muscle after each treatment, which might be one reason for its lower effectiveness.

In contrast to the modalities of PT (e.g., manual therapy, exercise, and TENS), which were independent and additive, all three elements of NT were found to be indispensable based on thousands of observations during its development. Thus, the objective of this study was to investigate the overall efficacy of NT and to compare it with the collective best practices of conventional PT. We did not intend to analyse individual elements/modalities of any therapy in this study.

Neither waveform nor frequency of the electro-stimulation contributed to the difference in efficacy between NT and PT-TENS. Prior studies concluded that different waveforms of TENS did not affect analgesic efficacy^34^. Thus, various waveforms were used in the PT-TENS arm. Moreover, the effect of NT was not affected by waveform. Both high (typically 80–150 Hz) and low (typically 1–10 Hz) frequencies were used in the PT-TENS arm in Studies One and Two without any noticeable impact on the outcomes at follow-up. Although low frequencies were used more often in the NT arm in this study, high frequencies were also used. The effect of NT was not affected by the stimulation frequency. High- and low-frequency TENS activate δ- and μ- opioid receptors, respectively, to cause only short-term analgesia^35^; thus, the frequency range was not responsible for the long-term effect of NT at follow-up. In this study, the waveform and the frequency were standardized in the protocol to facilitate the training of therapists and the clinical application of NT.

Depending on the severity and extensiveness of the pain, various neck (or shoulder) muscles could be involved. In severe cases, the Noxipoints of nearly all muscles in neck and shoulder regions were identified and treated. In milder cases, some patients had been treated at the Noxipoints related to less than ten muscle groups. In this study, muscle groups treated in most patients included trapezius, splenius capitus, rectus capitus, levator scapulae, supraspinatus and deltoid.

Often used to characterize the myofascial pain syndrome, Trigger Points (TrP, also called Myofascial Trigger Points) are defined as the tender (hyperirritable) spot in a palpable taut band of skeletal muscle fibers^36^. Pressure stimulation of a TrP can elicit pain, referred pain, and local twitch response. Some TrPs, similar to Noxipoints, are found on the attachments (“Attachment TrPs”). The distinctive characteristics of Noxipoints are that they (1) come in pair for each muscle or soft tissue, (2) precisely approximate the two attachments of the muscle group or soft tissue and (3) do not require the presence of taut band nor twitch response for identification. TrP treatments (such as saline injection) focus on each TrP, while NT stimulates the pair of Noxipoints in the same electro-circuit to elicit C-fiber-like sensation simultaneously. If the NT stimulation is applied to one Noxipoint only or if the pair of Noxipoints is not in the same close circuit, there is little effect.

The microstructure of Noxipoints in each muscle group is assumed to be the receptive endings of nociceptors (the receptors for potentially damaging stimuli that cause the pain sensation), which have been observed to be concentrated near the two attachments^30^. The facts that the density of nociceptor fibres in the peritendineum (the connective tissue around a tendon) of the rat’s tendon is multiple times higher than that in the muscle proper, and that the muscle proper is usually not supplied by any free nerve endings (including the nociceptive endings)^37^ are consistent with this assumption.

The proposed mechanism underlying the effects of NT departs from the conventional wisdom about chronic pain at the clinical and physiological levels. Although TENS was used in both the NT and PT-TENS arms, NT was distinct from conventional TENS with respect to its site specificity, submodality specificity and requirement for rest/immobilisation. Only induced soreness/dull pain consistently resulted in pain relief and persistent functional recovery. As the location of NT stimulation had to be at or near the two attachments of the target muscle, which contain the highest concentrations of nociceptors^30^, and as soreness/dull pain is the characteristic sensation elicited by C-fibres^38^, the C-fibre nociceptor was implicated in the NT process.

The sustained effect of substantial pain reduction and functional restoration in nearly all patients after NT could not be explained by the initial assumption that NT neurologically inhibited pain (e.g., via the activation of opioid receptors). The sustained effect, especially in patients with a history of muscle impairment, indicated that the outcome of NT involved muscle/tissue remodelling. The observed requirement for multiple-days of rest of the treated area for full functional recovery suggested the occurrence of cellular regeneration^32^.

Although the exact mechanism underlying its effects is unknown, a neuroimmune signalling pathway from the nociceptive signal to the innate immune cell-mediated repair process may explain the consistent outcome induced by NT stimulation^32^,^39^,^40^,^41^. Based on the biomedical literature, a specific induced signalling pathway has been hypothesised, and physiological research is underway to verify the involvement of this pathway. The coinciding outcome of the preliminary rat study may facilitate such research.

Though these two trials and the animal study provided evidence for a promising therapy, longer-term and broader-scope studies of multiple chronic pain conditions are desirable. Further studies of the individual elements of NT are also desirable. Detailed activity monitoring after treatment may be helpful to establish the precise correlation between rest (immobilisation) of the treated area and the effect of NT. Further animal studies would be useful for investigating the mechanism underlying the effect of NT.

These studies found that NT substantially reduced pain, restored function, and improved quality of life in a sustained manner in patients with chronic neck and shoulder pain and that NT was superior to conventional PT. Study Two found that the effects of NT are generalisable with respect to different pain levels, ethnicities and therapists.

## Methods

### Trial design

Study One was a parallel, blinded superiority RCT with crossover comparing the effectiveness of NT to that of PT-TENS in patients with chronic neck and shoulder pain. The trial consisted of two stages. The patients in Study One were first stratified based on the Brief Pain Inventory^42^ (BPI) “at its worst” (BPI ≥ 7.5 vs. BPI < 7.5) and then allocated at a 2:1 ratio using a computerised random-number generator to the test (or NT) arm, which received NT, and the control (or PT-TENS) arm, which received PT including TENS. The 2:1 randomisation ratio was used for ethical reasons due to the positive therapeutic effect of NT observed in preliminary treatments (Exhibit 3, Supplementary Protocol 1). A person at a separate location from the trial site retained and concealed the generated sequence. Qualified patients who verbally consented on the phone were assigned a sequential number. The scheduling administrator, who had no knowledge of the random allocation sequence, sequentially assigned each patient who signed the informed consent form to a stratum based on the reported pain level. The assignment of the patient was implicitly locked in at this step. Within each stratum, a person not involved in the trial mechanically mapped patients in the sequence to the pre-computed random allocation sequence to avoid assignment bias.

In the second stage, patients were allowed to cross over to the other arm after completion of treatment in the assigned arm, followed by a washout period (minimum of 4 weeks). At both stages, patient outcomes were evaluated at baseline, at each treatment session, and at follow-up, which was performed approximately 8 weeks after baseline (at least 4 weeks after the final treatment). Note that this crossover design eliminated any potential assignment/selection bias and other known or unknown confounders, which was a key objective of the trial design.

Study Two, a parallel, blinded superiority RCT, was conducted to compare the effectiveness of NT to that of PT-TENS for chronic neck pain using the same measures as those used in Study One and following up at 4 and 8 weeks after baseline assessment. The sequence for random allocation to either arm was generated using a computerised random number generator at a 1:1 ratio and was stored by a clerk who was not involved in the trial for concealment. A scheduling administrator, who had no knowledge of the random allocation sequence, assigned a sequential number to each patient who had signed the informed consent form. Each patient was mechanically mapped to the pre-computed allocation sequence by an independent person to avoid assignment bias.

All experiments were performed in accordance with relevant guidelines and regulations. Study One was approved by the Alpha Independent Review Board. Study Two was approved by the Research Ethics Committee of National Taiwan University (NTU) Hospital. Both trials were registered in www.clinicaltrials.gov (NCT01578148 on April 13, 2012 and NCT01844167 on April 22, 2013, respectively) and conformed to the CONSORT flowchart (Fig. 1) and checklist. Written informed consent was obtained from each participant prior to conducting any study procedures. The methods of both studies were similar; the detailed differences between the studies are presented in Table 4. The animal study was approved by the Institutional Animal Care and Use Committee of National Taiwan University (Approval No. NTU-103-EL-69). The study adhered to guidelines established by the Codes for Experimental Use of Animals from the Council of Agriculture of Taiwan, based on the Animal Protection Law of Taiwan.

The trial design followed the methodology recommended for chronic pain trials^2^,^26^.

### Patient selection

The eligibility criteria of Study One included age of 18 to 64 years, 6-month history of chronic neck pain (ICD-9: 723.1) or shoulder pain (ICD-9: 719.41), at least one prior pain treatment, and BPI “at its worst” ≥5. Neck and shoulder pain were selected because they were often comorbid. The primary exclusion criteria were traumatic bone injury secondary to external forceful impact and pain caused by traumatic bone fractures. The eligibility criteria of Study Two were age of 20 to 70s, 6-month history of neck pain, presence of trigger points, and BPI “at its worst” ≥5. The primary exclusion criteria included cervical spondylosis with imaging evidence of nerve root compression. Detailed patient selection criteria for both studies and the settings and locations in which the data were collected are described in the Patient Selection section of Table 4.

### Interventions

**Figure 3:**
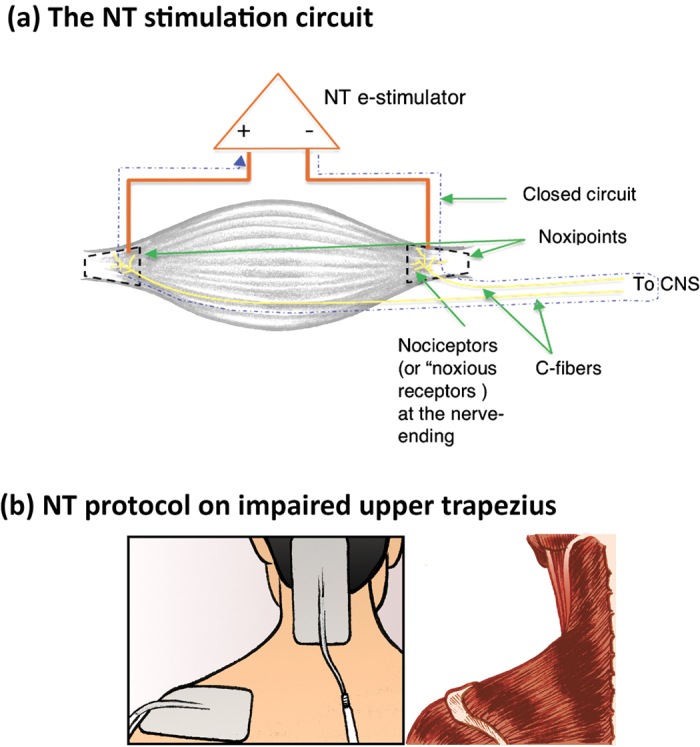
Application of NT. (**a**) The NT stimulation circuit. **(b**) NT protocol on impaired upper trapezius. A muscle group or soft tissue was identified as a target when Noxipoints™ appeared at both of its attachments as shown in (**a**). Stimulation was precisely applied at the pair of Noxipoints corresponding to the impaired muscle/tissue. Most chronic neck- or shoulder-pain patients had multiple groups of impaired muscles/tissues. An example of applying NT to the upper trapezius is shown in (**b**). The illustrations were drawn by Jesse Stark.

**Figure 4:**
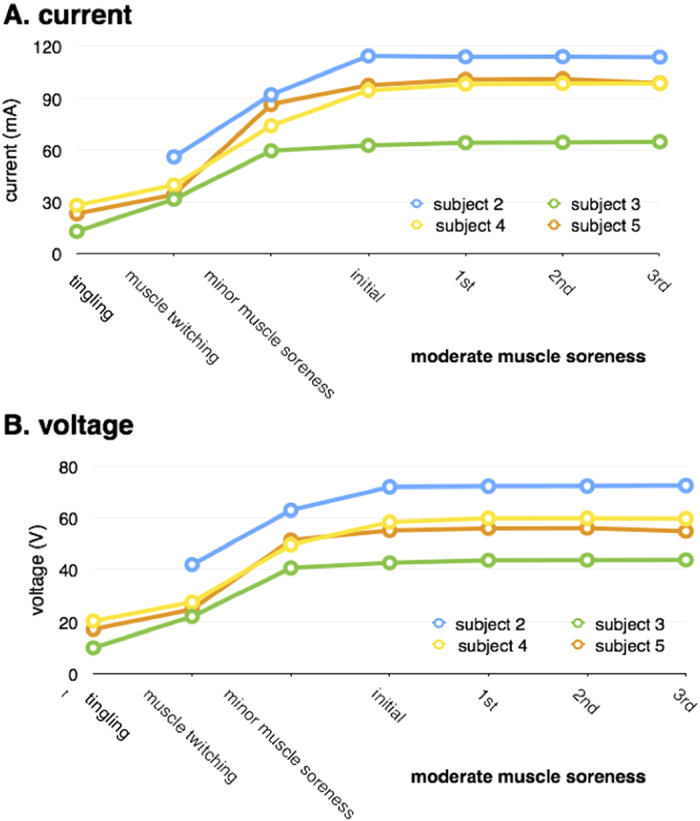
NT Stimulation Settings. Electrical stimulation was applied to the two attachments of the target muscle (e.g., deltoid) in NT. Different subjects responded differently to the current and the voltage used to attain the target for the nociceptive submodality (i.e., moderate muscle soreness/dull pain). The mode and the frequency were held constant for all subjects. Once the target nociceptive submodality was reached, the setting was maintained for approximately 4 minutes or until the corresponding Noxipoints™ disappeared. Muscle twitching/contraction and motor-nerve activation often preceded the onset of soreness during NT stimulation. The timeline shows the sequential appearance of a motor neuron response (“muscle contraction”), an A_β_ fibre response (“tingle”), and a C-fibre response (“muscle soreness”). An A_δ_ fibre response (sharp/spiky pain) was avoided by slowly and carefully increasing the intensity. The progression of different patients followed a common pattern but reached the nociceptive submodality at different voltage/current settings for each patient. The intensity (current or voltage) of “moderate muscle soreness” was approximately twofold that of “muscle twitching”/contraction. NT resolved the pain in the treated area when soreness was perceived in the presence or absence of muscle contraction.

Between 2012 and 2014, in Study One (Study Two), the patients in both arms received three (six) 90-minute treatment sessions within approximately 4 weeks. The patients in the NT arm were treated with electrical stimulation for 90 minutes according to the following guidelines described below. In the pain area, the therapist palpated at or near the attachment points of each muscle group and soft tissue and identified a set of pain points that were sensitive to pressure (termed Noxipoints™). A muscle group or soft tissue was identified as a target when Noxipoints appeared at both of its attachments. Stimulation was precisely applied at the pair of Noxipoints corresponding to the impaired muscle/tissue (Fig. 3) for approximately four minutes per application. Typical of most chronic neck- or shoulder-pain patients, most subjects had multiple groups of impaired muscles/tissues; a new pair of target Noxipoints was identified for the next stimulation. The stimulation was set up to induce the specific nociception of soreness or dull pain, but not sharp pain, based on the patient’s confirmation during the application. Stimulus voltage, wavelength, frequency and mode were collectively adjusted to achieve the target sensation. A typical range of the settings used for NT was as follows: mode, burst; pulse frequency, 1–3 Hz; pulse duration, 50–300 milliseconds; application duration, four minutes; intensity, 30–120 mA; and electrode characteristics, 2″ × 4″ reusable type or, occasionally, 2″ × 2″ for small muscle groups. Skin preparation was typically not necessary, but electrolyte solution or water was sprayed on dry skin to prevent electrical arcing. As shown in Fig. 4, the actual settings were measured and shown to illustrate multiple patients’ responses in sequence. Different subjects responded differently to the current and the voltage used to attain the target for the nociceptive submodality (i.e., moderate muscle soreness/dull pain). The mode (burst) and the frequency (2 Hz) were held constant for all subjects. Various combinations of parameters can be used to reach the target for the nociceptive submodality.

Within the 90-minute session, the objective was to apply NT to as many pairs of Noxipoints as possible; approximately 4 minutes were required for each NT application. The balance of the session time not including the stimulation time was used for positioning and tuning (which involved locating the Noxipoints, placing the pads, slowly adjusting the intensity to avoid a sudden increase (which could trigger A_δ_ fibre-elicited sharp pain) and obtaining feedback from the patient before each stimulation. The time required for these activities naturally varied (about 2–4 minutes), as each person was different. The “2–5 minutes” of stimulation in the original protocol was later standardised to 4 minutes per stimulation during NT application. The net outcome of NT was not sensitive to the duration of each stimulation, as the total session time remained the same (approximately 90 minutes). After each treatment session, the patients were instructed not to strain the newly treated muscle or tissue during the “resting period”, which was approximately 3 to 7 days, and to use braces in moderate or severe cases of pain (see the details of the Intervention section of Table 4).

The patients in the PT-TENS arm were treated for 90 minutes per session with conventional PT, including manual therapy (to cervical and/or rotator cuff areas), infrared heat therapy (on the pain site) and ROM exercises (foraminal opening, walking the arm on the wall, rotation of the upper arm, etc.) for 15 minutes each in Study One (20 minutes each in Study Two). TENS was applied to general pain areas identified by the patient and reoriented once for 45 minutes in Study One (30 minutes in Study Two). In both studies, the physical therapist was commissioned to optimise the treatment for individual patients within the protocol. In Study One, TENS application was set up as follows: mode, normal; frequency, 10–90 Hz; duration, 200–250 microseconds; waveform, pulsed biphasic, asymmetric square wave; input voltage, 9 V; output voltage: 30–90 V, intensity, maximum tolerable by the patient (typically 30 mA–60 mA) up to a maximum available current of 120 mA. In Study Two, TENS application was set up as follows: (a) mode, duration modulation; frequency, 80–100 Hz; duration, 100–150 microseconds; waveform, pulsed biphasic, asymmetric square wave; input voltage, 9 V; output voltage, 30–80 V; and intensity, maximum tolerable by the patient (typical 15–60 mA) up to a maximum available current of 120 mA; (b) mode, strength-duration modulation 2 (SD2); frequency, 5–60 Hz; duration, 40–150 microseconds; waveform, variable pulses with automatic increasing intensity and decreasing pulse duration; input voltage, 9 V; output voltage, 30–100 V; intensity, maximum tolerable by the patient up to a maximum available current of 120 mA. TENS application was set at the highest tolerable intensity and frequency based on the patient’s feedback as recommended in prior studies^43^. As the physical therapist was asked to optimise the PT-TENS treatment for the patient, alternative TENS settings might have been used when little progress was observed during the first several sessions (interferential current mode with crossed channel placement; frequency, 4000–4100 Hz with 70–120 Hz differential; duration, 50–100 microseconds, waveform, sine wave; intensity, maximum tolerable by the patient (typically 15–60 mA) up to a maximum available current of 99 mA). Following best PT practices, the patients were asked to follow self-care instructions including maintaining good posture (e.g., sitting up straight) throughout the day, performing stretch exercises (e.g., chin in, chin in & slowly stretch head back, head turn over the shoulders, shoulder rolls, shoulder blade squeeze, 10 times each) and endurance training and re-educating muscles.

No skin preparation was needed for NT or PT in most cases. An electrolyte solution or water was occasionally sprayed on the dry skin or near the hairline to avoid electrical arcing.

One licensed physical therapist in each study with a minimum of 12 years of work experience and no knowledge of NT performed the PT-TENS. After the PT-TENS protocol was explained to the physical therapist, he/she was commissioned to optimise outcomes for each patient within the protocol. NT was performed by the original therapist team in Study One and by three newly trained therapists in Study Two. Each new NT therapist was trained via an NT introduction section for four hours by the original therapist followed by approximately 20 practical training sessions (approximately 4–5 patients) before the new therapist began treating patients on her/his own to ensure consistency in locating Noxipoints and performing treatments. The six sessions allocated in Study Two, representing a standard physician’s order for any PT, partially accommodated the inexperience of the NT therapists.

The selection of controls was based on multifaceted considerations. Nearly all patients with long-term chronic pain had undergone multiple treatment modalities, including manual therapy, active use of TENS, observation (no treatment), waiting, etc. for years. Although placebo TENS (placing electrodes on sites without applying current) was considered, this treatment was unlikely to pass the sanity check of any patient. Thus, we included TENS with current in the control arm to blind the patient to the treatment. Between TENS with random placement and TENS following conventional PT guidelines, we chose the latter to maximise the effect of the control arm, erring on the conservative side. That is, TENS in the PT-TENS arm was not a sham therapy but rather a component of active treatment.

Although little evidence suggested that any specific modality of PT had greater long-term efficacy than placebo, significant evidence showed that most modalities of PT, including placebo, “were almost always better than no treatment in treating chronic pain”^44^. PT, especially PT using multiple modalities including TENS, was therefore an ethical and robust control for this study.

The objective of the study was to investigate the overall efficacy of NT and to compare it with the best practice of conventional PT (i.e., the standard of care) as an established active comparator. We did not intend to determine which element in NT or modality in PT-TENS contributes more (or less) to treatment efficacy. Thus, multiple modalities of PT with established efficacy were included in PT-TENS as the best-practice active comparator.

### Outcome measures

The primary outcome was the BPI score “at its worst”. The four secondary outcome measures were the BPI scores “average” and “right now”, functional impairment (defined by active pain-free ROM of six cervical and five shoulder dimensions, Supplementary Table 1) and QOL (measured by the BPI “interference with functions”; see details in the Outcome Measures section of Table 4). All BPI measures were reported by patients on the BPI Short Form (BPI-SF), and ROM was evaluated by the assessor and confirmed by the patient.

All measures were evaluated before each treatment session and at follow-up with the exception of ROM in Study Two, which was measured only before the first session and at follow-up. The BPI “right now” was also evaluated after each session. Every cancellation was followed up to determine whether it was a successful early completion or a dropout.

The reliability and the validity of BPI (and BPI “at its worst” in particular) have been established for chronic pain^45^,^46^,^47^. The reliability and the validity of a goniometer for measuring ROM has been established^48^.

### Blinding

To ensure patient blinding, TENS was included in both arms; the technical difference in TENS application between NT and PT-TENS was not described to the patient *a priori*. Investigators who interacted with patients were trained not to compare the differences in expected benefits to avoid a psychological placebo effect^2^.

The assessment of BPI pain subtypes and interference with functions was blinded, as the patients reported the BPI scores. The assessment of ROM was objectively performed by the provider using a goniometer and was confirmed by the patient in Study One and by a third-party assessor in Study Two.

### Statistical methods

The primary analysis compared all outcome measures at follow-up to baseline for each arm at each stage using t tests and Wilcoxon signed-rank tests. Differences in these outcome measures were further compared using t tests and Wilcoxon rank-sum tests between the NT arm and the PT-TENS arm during the first stage and between the crossover-to-NT and PT-TENS patients during the second stage. Because there were only two time points for the outcome measures in the primary analysis, repeated-measures analyses such as repeated-measures ANOVA were not applicable. All comparisons were tested at the two-sided significance level of 5% (i.e., p < 0.05). The secondary analysis in Study One further evaluated the incremental changes in these outcome measures during the treatment course. The statistical analysis software R (version 2.15) was used in Study One, and both R and SAS were used in Study Two.

All analyses were performed according to the originally assigned arms. Missing data were imputed using last-observation-carried-forward (LOCF). Patients not receiving any post-baseline assessment were excluded. All randomised patients who received any treatment (even only one session) and one post-treatment assessment were included in the analysis to avoid potential bias introduced by excluding dropout patients. The missing outcome measures for these dropout patients (e.g., 5 patients lost to follow-up in the NT arm of Study Two) were conservatively imputed using LOCF. Thus, the number of patients analysed was greater than the number of patients observed at follow-up in both studies. All Ns are reported in the CONSORT Flowchart (Fig. 1). All patients who received additional, off-protocol treatments (particularly those in the PT-TENS arm) were included in the analysis to be conservative.

Patients with successful early completion (i.e., no further treatment sessions needed) reported their pain level and QOL by phone; the ROM measures had to be assessed in person and were thus missing for these sessions. These missing interim ROM measures used in the secondary analysis were imputed backward based on the measures observed at follow-up (presumably the worst observation).

### Sample size

The effective sample size (before dropout was considered) in Study One was 28 based on the outcome of the preliminary study (Exhibit 3; Supplementary Protocol 1). We planned to enrol approximately 35 patients (at a 2:1 randomisation ratio of the NT arm to the PT-TENS arm). This sample size provided 90% power to detect a 4-point mean difference between the two arms in BPI “at its worst” from baseline (i.e., the effect size) at a two-sided significance level of 5%, assuming a standard deviation of the change from baseline in BPI “at its worst” in both arms of 3 points and a dropout rate of approximately 20%.

The effective sample size (before dropout was considered) in Study Two was 44 based on the outcome of Study One. The plan was to enrol up to 80 patients (with a 1:1 randomisation ratio of the NT arm vs. to the PT-TENS arm). This sample size was expected to provide 90% power to detect a 3-point mean difference between the two arms in BPI “at its worst” from baseline (due to the reduced NT effect expected from newly trained therapists) at a two-sided significance level of 5%, assuming a standard deviation of the change from baseline BPI “at its worst” in both arms of 3 points and a conservative dropout rate of approximately 40% (due to local experience). Note that the actual dropout rate in Study Two was less than expected.

The enrolment size of Study Two was larger than that of Study One because (1) the assumed difference of the effect size in the two arms was expected to be smaller in Study Two due to the enrolment of less heavily pretreated patients (who may have also responded to PT), (2) the assumption that newly trained NT therapists would be less effective, and (3) the higher expected dropout rate due to the high accessibility of the public healthcare system in Taiwan.

### Animal study

Based on the outcome of NT-like treatment in a previous pilot rat study, 18 rats were subjected to induced mechanical hypersensitivity in the hind paw via repeated acidic saline injections (pH = 4, twice, five days apart) to the gastrocnemius muscle according to standard protocols^24^,^49^. These rats were randomly assigned to three groups: a control group that received sham therapy (6 rats, electrodes attached, no stimulus), a test group receiving NT-like treatment once (6 rats, NT-like treatment for 3 minutes) and another test group receiving NT-like treatment twice (6 rats, NT-like treatment for 3 minutes twice within three days). We periodically measured the sensitivity of the hind paw before and after treatment^49^,^50^ for 30 days (see detailed methods in Supplementary Text 3).

### Data and materials availability

The original study protocols and the full datasets used to produce the results are included as Supplementary data files. Informed patient consent was obtained and the presented data are anonymised and risk of identification is low. No image that identifies a patient was presented.

## Additional Information

**How to cite this article**: Koo, C. C. *et al.* Novel Noxipoint Therapy versus Conventional Physical Therapy for Chronic Neck and Shoulder Pain: Multicentre Randomised Controlled Trials. *Sci. Rep.* **5**, 16342; doi: 10.1038/srep16342 (2015).

## Supplementary Material

Supplementary Materials

Supplementary Dataset 1

Supplementary Dataset 2

Supplementary Dataset 3

## Figures and Tables

**Table 1:** Patient Characteristics at Baseline.

	Study One		Study Two	
	NT	PT-TENS	NT	PT-TENS
N at randomisation	24	10	23	21
Age	48 (±8.2)^a^	48 (±4.1)	41.7 (±6.3)	46.9 (°10.8)
Gender (female %)	14 (70.8%)	8 (80%)	14 (61%)	19 (91%)
Diagnosis (distribution)				
Neck pain (ICD-9: 723.1)	18 (75%)	8 (80%)	23	21
Shoulder pain (ICD-9: 719.41)	6 (25%)	2 (20%)	N/A	N/A
Primary pain, in BPI “at its worst”^b^	7.7 (±0.9)	8.1 (±1.1)	6.6 (±1.3)	6.4 (±1.4)
≥7.5 (number of patients (%))	15 (62.5%)	6 (60%)	N/A	N/A
<7.5 (number of patients (%))	9 (37.5%)	4 (40%)	N/A	N/A
Accum. functional impairment score	10.2 (±6.9)	5.8 (±8)	14.1 (±9.1)	14.8 (±6.6)
QOL, Accumulative “BPI “interference with functions”	26.4 (±16.6)	27.5 (±13.3)	36.3 (±12.0)	33.4 (±12.9)
Pain history, years	6.7 (±5.6)	6.7 (±4.6)	>0.5	>0.5
Prior treatment received (Number of distinct types of treatment)^c^	2.6 (±1.1)	3.0 (±1.2)	Not required	Not required
Prior treatment, number of patients				
Acupuncture	18 (75%)	5 (63%)	Not required	Not required
Massage	17 (71%)	7 (88%)	Not required	Not required
Physical therapy	12 (50%)	1 (13%)	Not required	Not required
Chiropractic	7 (29%)	2 (25%)	Not required	Not required
Analgesic	5 (21%)	5 (63%)	Not required	Not required
Surgery	1 (4%)	0 (0%)	Not required	Not required
Corticoid injection	1 (4%)	0 (0%)	Not required	Not required
Myofascial therapy	0 (0%)	1 (13%)	Not required	Not required
N analysed, with at least one post-baseline assessment^e^	22	8	20	18
N with follow-up 1 assessment^f^	19	8	16	18
N with follow-up 2 assessment^f^	19	8	16	17
Wait/washout period before follow-up (FU), in weeks (min, max)^g^	7 (4, 17)	5 (2, 7)	Pre-FU1: 2.5 (0.4, 10.3) Pre-FU2: 9.7 (4.0, 19.7)	Pre-FU1: 2.3 (0.3, 11.4) Pre-FU2: 6.7 (4.7, 9.7)

^a^Mean (±SD).

^b^The baseline difference between the NT and the PT-TENS patients was not statistically significant.

^c^The length of each type of prior treatment varied. Some treatments lasted multiple months/years (e.g., PT, acupuncture).

^d^The wait period before Follow-ups 1 and 2 were at least 1 and 4 weeks after the final on-protocol treatment session, respectively.

^e^Two NT subjects did not return after Session 1 and did not respond to phone calls. The pain conditions of both subjects improved after Session 1. One PT-TENS subject dropped out Pre-Session 1 because the condition of the subject improved. Another PT-TENS subject dropped out after Session 1 and did not respond to phone calls.

^f^One NT subject who did not receive follow-up was pain-free based on the Pre-Session 2 assessment; this subject left town permanently. Two NT subjects did not return without explanation.

^g^In Study One, one PT-TENS subject insisted in crossing over to the other treatment two weeks after the final allocated PT-TENS session, citing no relief from pain. We initiated NT treatment sooner than the required washout duration for the benefit of the subject. The wait/washout periods before follow-up for the NT, PT-TENS and XO arms were 7 (min 4, max 17), 5 (min 2, max 7) and 6 (min 5, max 9) weeks, respectively.

**Table 2:** Differences in the Changes in the Primary and Secondary Outcomes.

A. Study One^a^,^c^					
Measurement	Mean of Change (SD)^b^ in NT (n = 22)	Mean of Change (SD) in PT-TENS (n = 8)	Difference of Change in NT vs. PT-TENS (range), 95% CI^b^	Two-Sample t Test p value	Wilcoxon Rank-sum test p value
a. NT versus PT-TENS					
BPI “at its worst”	−6.9 (1.7)	0.1 (1.6)	−7.0 (−8.5–−.6)	<0.0001	<0.0001
BPI “average”	−4.6 (1.7)	0.7 (1.3)	−5.3 (−6.6–−4)	<0.0001	<0.0001
BPI “right now”	−4.7 (2.5)	1.2 (1.3)	−6.0 (−7.4–−4.5)	<0.0001	<0.0001
Functional Impairment	−7.9 (6.6)	1.2 (5.7)	−9.2 (−14.4–−3.9)	0.0021	0.0012
Interference with functions	−23 (17.2)	0.4 (4.6)	−23.4 (−31.6–−15.2)	<0.0001	<0.0001
**Measurement**	**Mean of Change (SD) in XO (n = 7)**	**Mean of Change (SD) in PT-TENS (n = 8)**	**Difference of Change in XO vs. PT-TENS (95% CI)**	**Two-Sample t Test p value**	**Wilcoxon Rank-sum test p value**
b. XO versus PT-TENS					
BPI “at its worst”	−6.9 (1.8)	0.1 (1.6)	−7 (−8.9–−5)	<0.0001	0.0012
BPI “average”	−5.8 (1.6)	0.7 (1.3)	−6.5 (−8.1–−4.8)	<0.0001	0.0013
BPI “right now”	−6 (1.8)	1.2 (1.3)	−7.2 (−9–−5.4)	<0.0001	0.0014
Functional Impairment	−4.9 (2.9)	1.2 (5.7)	−6.1 (−11.2–−1.1)	0.022	0.017
Interference with functions	−25.4 (11.5)	0.4 (4.6)	−25.8 (−36.6–−15)	0.0006	0.0003
**B. Study Two**^d^					
**Measurement**	**Mean of Change (SD) in NT (n = 20)**	**Mean of Change (SD) in PT-TENS (n = 18)**	**Difference of Change in NT vs. PT-TENS (range), 95% CI**	**Two-Sample t Test p value**	**Wilcoxon Rank-sum test p value**
a. NT versus PT-TENS (at Follow-up 1)					
BPI “at its worst”	−4.5	−1.8	−2.7 (−4.2–−1.1)	0.0015	0.00093
BPI “average”	−3.6	−0.9	−2.8 (−4.0–−1.5)	<0.0001	0.00014
BPI “right now”	−3.0	−0.4	−2.6 (−3.9–−1.2)	0.0005	0.0009
Functional Impairment*	−8.1 (n = 16)	−3.6 (n = 17)	−4.5 (−8.9–0.1)	0.046	0.10
Interference with functions	−27.8	−11	−16.8 (−26.3–−7.2)	0.001	0.0018
b. NT versus PT-TENS (at Follow-up 2)					
BPI “at its worst”	−4.3 (2.2)	−2.0 (2.9)	−2.3 (−3.9–−0.6)	0.0095	0.016
BPI “average”	−3.5 (1.8)	−0.9 (2.1)	−2.6 (−3.9–−1.3)	0.0003	0.0009
BPI “right now”	−3.0 (1.9)	−0.1 (1.0)	−2.9 (−4.2–−1.7)	<0.0001	0.0002
Functional Impairment**	−7.2 (6.1) (n = 16)	−3.8 (5.9) (n = 17)	−3.4 (−7.7–0.9)	0.11	0.23
Interference with functions	−27.7	−11.3	−16.4 (−25.5–−7.2)	0.0009	0.0007

^a^The average numbers of sessions of NT, PT-TENS, and XO in Study One were 2.3, 2.9 and 2.1, respectively.

^b^SD, standard deviation; CI, confidence interval.

^c^No patient received off-protocol treatments in any arm in Study One.

^d^In Study Two, some PT-TENS patients received off-protocol treatment; some of these patients underwent the Follow-up 2 assessment during their off-protocol treatments.

**Table 3:** BPI Scores, Functional Impairment, and Quality of Life at Pre-Session 1 (Baseline) Compared to those at Follow-Up in the NT, PT-TENS and Cross-over-to-NT (XO) Groups.

A. Study One^a^,^b^					
Measurement	Mean Score Pre-Session1 (SD)	Mean Score at Follow-Up (SD)	Mean of Score Difference (95% CI)	One-Sample t test p-value	Wilcoxon Signed-rank testp-value
BPI “at-its-worst”					
NT (n = 22)	7.7 (0.9)	0.8 (1.5)	−6.9 (−7.7–−6.1)	<0.0001	<0.0001
PT-TENS (n = 8)	8.1 (1.1)	8.2 (1.5)	0.1 (−1.2–1.5)	0.84	1
XO (n = 7)	8.1 (1.6)	1.3 (1.1)	−6.9 (−8.5–−5.2)	0.00059	0.022
BPI “average”					
NT (n = 22)	5.2 (1.5)	0.5 (1.2)	−4.6 (−5.4–−3.9)	<0.0001	<0.0001
PT-TENS (n = 8)	5.9 (1.5)	6.6 (1.7)	0.7 (−0.4–1.8)	0.19	0.20
XO (n = 7)	6.4 (1.7)	0.6 (0.7)	−5.8 (−7.2–−4.3)	0.00069	0.022
BPI “right-now”					
NT (n = 22)	5.2 (2.3)	0.6 (1.3)	−4.7 (−5.8–−3.5)	<0.0001	<0.0001
PT-TENS (n = 8)	5.6 (2.2)	6.8 (2)	1.2 (0.1–2.3)	0.037	0.58
XO (n = 7)	6.6 (2.1)	0.6 (0.5)	−6 (−7.7–−4.3)	0.00013	0.022
Functional Impairment					
NT (n = 22)	10.2 (6.9)	2.3 (3.3)	−7.9 (−10.8–−5)	<0.0001	<0.0001
PT-TENS (n = 8)	5.8 (8)	7 (4.9)	1.2 (−3.5–6)	0.55	0.59
XO (n = 7)	6.7 (5.2)	1.9 (2.9)	−4.9 (−7.5–−2.2)	0.0045	0.022
Quality of Life- Accumulative BPI “interference with functions”					
NT (n = 22)	26.4 (16.6)	3.4 (8)	−23 (−30.6–−15.4)	<0.0001	<0.0001
PT-TENS (n = 8)	27.5 (13.3)	27.9 (10.9)	0.4 (−3.4–4.3)	0.80	0.83
XO (n = 7)	29.1 (11.3)	3.7 (5.6)	−25.4 (−36–−14.7)	0.0011	0.015
**B. Study Two**					
**Measurement**	**Mean Score Pre-Session1 (SD)**	**Mean Score at Follow-1 (SD)**	**Mean of Score Difference (95% CI)**	**One-Sample t Test p-value**	**Wilcoxon Signed-rank testp-value**
(a) Baseline vs. Follow-up 1					
BPI “at-its-worst”					
NT(n = 20)	6.5 (1.3)	2.1 (2.0)	−4.5 (−5.6–−3.3)	<0.0001	0.0001
PT-TENS (n = 18)	6.4 (1.5)	4.1 (1.8)	−1.8 (−3.0–−0.6)	0.0058	0.0039
BPI “average”					
NT (n = 20)	5.0 (1.4)	1.3 (1.4)	−3.6 (−4.5–−2.8)	<0.0001	0.00013
PT-TENS (n = 18)	4.3 (1.4)	3.4 (1.5)	−0.9 (−1.8–−0.1)	0.064	0.05
BPI “right-now”					
NT (n = 20)	4.3 (1.6)	1.4 (1.5)	−3.0 (−4.0–−2.0)	<0.0001	0.00022
PT-TENS (n = 18)	3.3 (2.2)	2.8 (1.5)	−0.4 (−1.5–0.6)	0.37	0.28
Functional Impairment					
NT	14.1 (9.1) (n = 20)	7.6 (8.4) (n = 16)	−8.1 (−11.5–−4.6)	0.00018	0.001
PT-TENS	14.8 (6.6) (n = 18)	11.6 (6.1) (n = 17)	−3.6 (−6.5–0.6)	0.02	0.027
Quality of Life- Accumulative BPI “interference with functions”					
NT (n = 20)	36.3 (12.0)	8.6 (10.9)	−27.8 (−34.3–−21.2)	<0.0001	0.0001
PT-TENS (n = 18)	33.4 (12.9)	22.4 (10.9)	−11 (−18.4–−3.6)	0.0058	0.01
(b) Baseline vs. Follow-up 2					
BPI “at-its-worst”					
NT(n = 20)	6.5 (1.3)	2.3 (1.8)	−4.45 (−5.6–−3.3)	<0.0001	<0.0001
PT-TENS (n = 18)	6.4 (1.5)	4.4 (2.2)	−2.3 (−3.7–−0.9)	0.0040	0.0054
BPI “average”					
NT (n = 20)	5.0 (1.4)	1.5 (1.5)	−3.5 (−4.4–−2.6)	<0.0001	<0.0001
PT-TENS (n = 18)	4.3 (1.4)	3.3 (1.9)	−0.9 (−2.0–−0.1)	0.007	0.08
BPI “right-now”					
NT (n = 20)	4.3 (1.6)	1.4 (1.4)	−3.0 (−3.9–−2.1)	<0.0001	<0.0001
PT-TENS (n = 18)	3.3 (2.2)	3.2 (1.8)	−0.06 (−1.0–0.9)	0.9	0.9
Functional Impairment					
NT	14.1 (9.1) (n = 20)	8.9 (8.2) (n = 15)	−6.9 (−10.3–−3.5)	0.0007	0.0005
PT-TENS	14.8 (6.6) (n = 18)	11.6 (5.8) (n = 16)	−3.6 (−6.9–−0.4)	0.03	0.02
Quality of Life- Accumulative BPI “interference with functions”					
NT (n = 20)	36.3 (12.0)	8.7 (9.0)	−27.7 (−33.9–−21.4)	<0.0001	<0.0001
PT-TENS (n = 18)	33.4 (12.9)	22.1 (13.5)	−11.3 (−18.4–−4.1)	0.004	0.006

^a^All results for the NT (test arm), PT-TENS (control arm) and XO (Crossover-to-NT) groups followed LOCF imputation. All patients except those undergoing no post-baseline observations were included.

^b^SD, standard deviation; CI, confidence interval.

**Table 4:** Comparison of the Methods used between Studies One and Two.

Methods	Study One–RCT in California	Study Two- RCT in Taiwan
**Design**	Prospective, two-arm, patient- and assessor-blinded, stratified RCT with crossover.	Prospective, two-arm, patient- and assessor-blinded RCT.
	Stratified according to BPI≥7.5 or <7.5. Randomised at a 2:1 ratio (NT: PT-TENS). Crossover after a washout period (minimum of 4 weeks).	Randomised at a 1:1 ratio.
**Patient selection**	April 2012 to Feb 2013	April 2013 to May 2014
	Recruitment: 2012/03/30-2013/01/04	Recruitment: 2013/05/10-2014/03/03
	34 patients consented & randomised.	44 patients consented & randomised.
Inclusion criteria:	Age 18 to 64 years, six-month history of chronic neck pain (ICD-9: 723.1) or shoulder pain (ICD-9: 719.41), received prior pain treatments, BPI “at its worst” ≥5.	Age 20 to 70 years, six-month history of chronic neck pain (ICD9: 723.1) with trigger points, BPI “at its worst” ≥5.
Exclusion criteria:	Traumatic bone injury secondary to external forceful impact, pain caused by traumatic bone fractures, local steroid injection within the last 4 weeks, history of traumatic cervical injury or osteoporosis, pain related to systemic inflammatory conditions including polymyalgia rheumatica and systemic lupus erythaematosis, signs of psychosomatic illness, severe rheumatoid arthritis under active treatment including disease-modifying antirheumatic drugs.	Cervical spondylosis (with imaging evidence of nerve root compression), local corticoid injection within the last two weeks, unwilling to be randomised, pregnant woman.
Settings and Locations:	Therapy and data collection were performed at the treatment room of the Pain Cure Center, Palo Alto, California.	Therapy and data collection were performed at an outpatient exam room of the Department of Physical Medicine and Rehabilitation, National Taiwan University Hospital, Taipei, Taiwan.
Note:	Pathology of the pain was not ascertained at baseline, as most severe chronic pain cases were idiopathic, but this characteristic was subsequently determined via patient interview or observation during treatment.	Muscle/soft tissue impairment at the neck was implicated by excluding cervical spondylosis.
Intervention	Treated for up to 3 sessions, 90 minutes per session.	Treated for up to 6 sessions, 90 minutes per session.
NT:	Palpated the attachment points of each muscle group and soft tissue in the pain area and identified a set of pain points sensitive to pressure (termed Noxipoints™). A muscle group or soft tissue was identified as a target when Noxipoints appeared on both of its attachments. A three-dimensional Noxipoint™ Navigation System was provided to help the NT therapist locate Noxipoints during the treatment. The stimulation pads were precisely placed at the skin surface locations of the pair of Noxipoints corresponding to the impaired muscle/tissue for approximately 4 minutes per application. As most chronic neck or shoulder pain patients had multiple groups of impaired muscles/tissues, a new pair of target Noxipoints was identified for the next pad placement and stimulation after each application. The positioning and tuning of each stimulation took approximately 2-4 minutes, as each patient reacted differently. From ten to fifteen stimulations were applied to the patient per session. Multiple pairs of Noxipoints were stimulated at the same time in some cases. The stimulation was set up to induce the specific nociception of soreness, achiness or dull pain based on the patient’s confirmation during the application. Stimulus voltage, wave pattern, wavelength, frequency and mode were collectively adjusted to achieve such sensations. Representative settings are presented in Fig. 4. After the treatment session, the patients were instructed not to strain the newly treated muscle or tissue during the “resting period” (described below) and to use braces in moderate or severe cases. The duration of the “resting period” was a minimum of three days for patients below 40 years old and one additional day per 10-year increment. For example, the rest duration for a 45-year-old patient was 4 days. This estimate was suggested based on the heuristic observation of a few hundred preliminary cases. If the patient had extensively impaired muscles, he/she was instructed to take two additional days of rest of the muscles. As all participants had moderate to severe pain, they were asked to use braces (e.g., OTC Professional Orthopedic Soft Foam Cervical Collar, Futuro Soft Cervical Collar, over-the-counter arm sling or tape) to immobilise the treated area.	NT was performed as in Study One
PT:	Manual therapy, infrared heat therapy and ROM exercises for 15 minutes each, and TENS for 45 minutes at the general pain areas identified by the patient; the pain areas were reoriented once.	Manual therapy, infrared heat therapy and ROM exercises for 20 minutes each, and TENS for 30 minutes at the general pain areas identified by the patient; the pain areas were reoriented once.
Outcome measures	BPI “at its worst”, “average”, and “right now” (each from 0 to 10), ROM deficit, and BPI interference with functions. The primary outcome was BPI “at its worst”. Active, pain-free ROM was measured for the neck in 6 dimensions (flexion, extension, lateral flexion to the right, lateral flexion to the left, rotation to the right, and rotation to the left) and for the shoulder in 5 dimensions (flexion, extension, internal rotation, external rotation, and abduction). The ROM in each dimension was mapped to a severity level (from 0 (normal) to 7 (immobile)) as shown in Supplementary Table 1. The patient’s ROM limitation severity levels in all dimensions were summed reflect the cumulative effect of the breadth and the depth of functional impairment.	The same as those in Study One.
	Patients reported the pain scales and the QOL measures on the BPI-SF.	Patients reported the pain scales and the QOL measures electronically online.
	Assessors evaluated ROM using a goniometer, and the results were confirmed by the patients. Device: US FDA #K080661	Third-party assessors evaluated ROM using a goniometer. US FDA #K071951 (Noxipoint e-Stim)
Assessment	Baseline, Before and after each session, 4 weeks after the final session (about 8 weeks after baseline), and 4 months after the final session on randomly selected patients.	Baseline, Before each session (pain scores and QOL), 4 weeks after baseline or 1 week after the final session, and 8 weeks after baseline.
Statistical analysis	The significance level was set to 0.05 (p<0.05). All estimated p-values were two-sided.	The same as Study One.
	Missing data were imputed using the LOCF method.	The same as Study One.
