# Supplementary material for: Novel Noxipoint Therapy versus Conventional Physical Therapy for Chronic Neck and Shoulder Pain: Multicentre Randomised Controlled Trials
Source: Sci Rep. 2015 Nov 10;5:16342. doi: 10.1038/srep16342 (PMC4639784; doi:10.1038/srep16342)
Supplement: Supplementary Materials [file srep16342-s1.doc]

**Supplementary Materials**

**Novel Noxipoint Therapy versus Conventional Physical Therapy**

**for Chronic Neck and Shoulder Pain: Multicentre Randomised Trials**

Charles C. Koo, PhD, Ray S. Lin, PhD, Tyng-Guey Wang, MD, Jau-Yih Tsauo, PhD, Pan-Chyr Yang, MD, PhD, Chen-Tung Yen, PhD, Sandip Biswal, MD

**Table of Contents Page**

| Supplementary Table 1 | 2 |
| --- | --- |
| Supplementary Figure 1 | 3 |
| Supplementary Figure 2 | 5 |
| Supplementary Figure 3 | 6 |
| Supplementary Text 1 | 8 |
| Supplementary Text 2 | 10 |
| Supplementary Text 3 | 11 |
| Supplementary Text 4 | 14 |
|  |  |

**Supplementary Table 1. Functional Impairment: Mapping of the Limitation of Range of Motion, Expressed as Severity and % of Full ROM**

| **ROM limitation** | **Severity level** | **% of full ROM** | | | |
| --- | --- | --- | --- | --- | --- |
| Normal | 0 | > | 90% |  |  |
| Mild | 1 | > | 80% |  |  |
| Moderate | 2 | > | 70% |  |  |
| Severe | 3 | > | 60% |  |  |
| Very severe | 4 | > | 50% |  |  |
| Handicapped | 5 | > | 40% |  |  |
| Severely handicapped | 6 | > | 30% |  |  |
| Immobile | 7 (max) | ≤ | 30% |  |  |

Functional impairment was obtained by summing the severity levels in all dimensions. For example, a patient who had scores of 3 (i.e., “severely impaired”) in two dimensions received a cumulative score of 6. Functional impairment of individual patients in Study One ranged from 0 to 42; however, these patients did not always have severe impairment in all dimensions.

A patient’s functional impairment was a composite index of the depth and the breath of the impairment; it was not necessarily proportional to the reported pain level, which was dictated by the most severe pain among all dimensions. The patient did not have to be severely limited in all dimensions to be in severe pain.

**Supplementary Figure 1. BPI “At Its Worst” for Individual Patients in Study One (for every patient who was assigned to the NT arm and assessed at follow-up)**


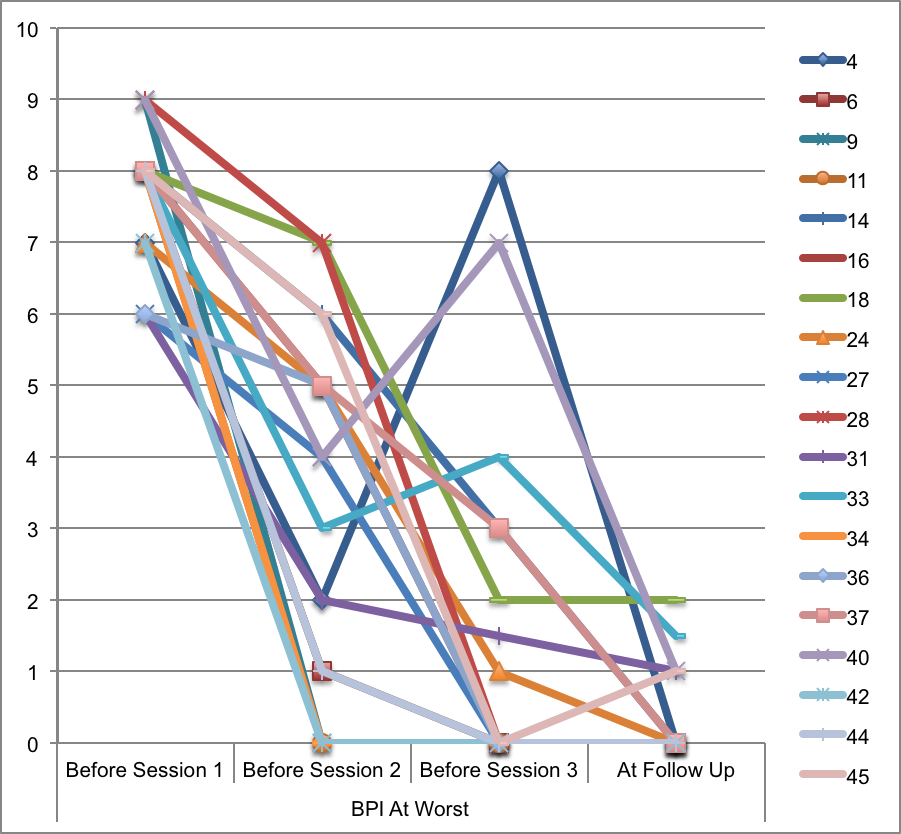


**Patient ID**

| 1. 4/19 patients reported a pain score of 0 (successful completion) within 1 session (21%).  2. 6/19 patients reported a pain score of 0 (successful completion) within 2 sessions (32%). |
| --- |
| 3. 4/19 patients reported a pain score of 0 (successful completion) within 3 sessions (21%).  4. 5/19 patients reported a nonzero pain score at follow-up; the pain score was ≤2 in all such cases. |

Average number of NT sessions in Study One = 2.26.

**Supplementary Figure 2. Incremental Pain Changes Due to NT for Selected Patients in the NT Arm**

**
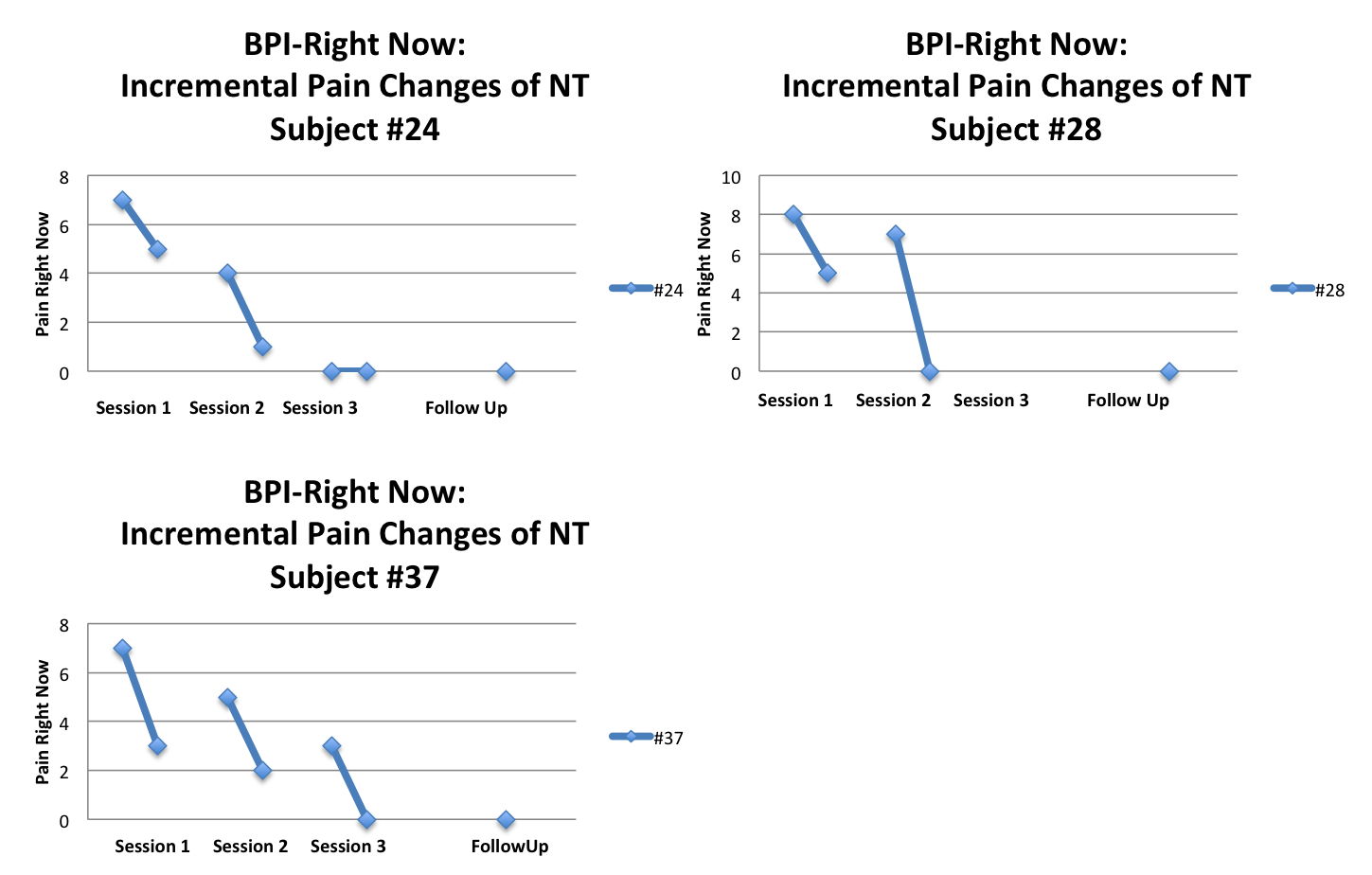
**

**Supplementary Figure 3. Changes In Chronic Hyperalgesia in the Hind Paw of Rats**

**
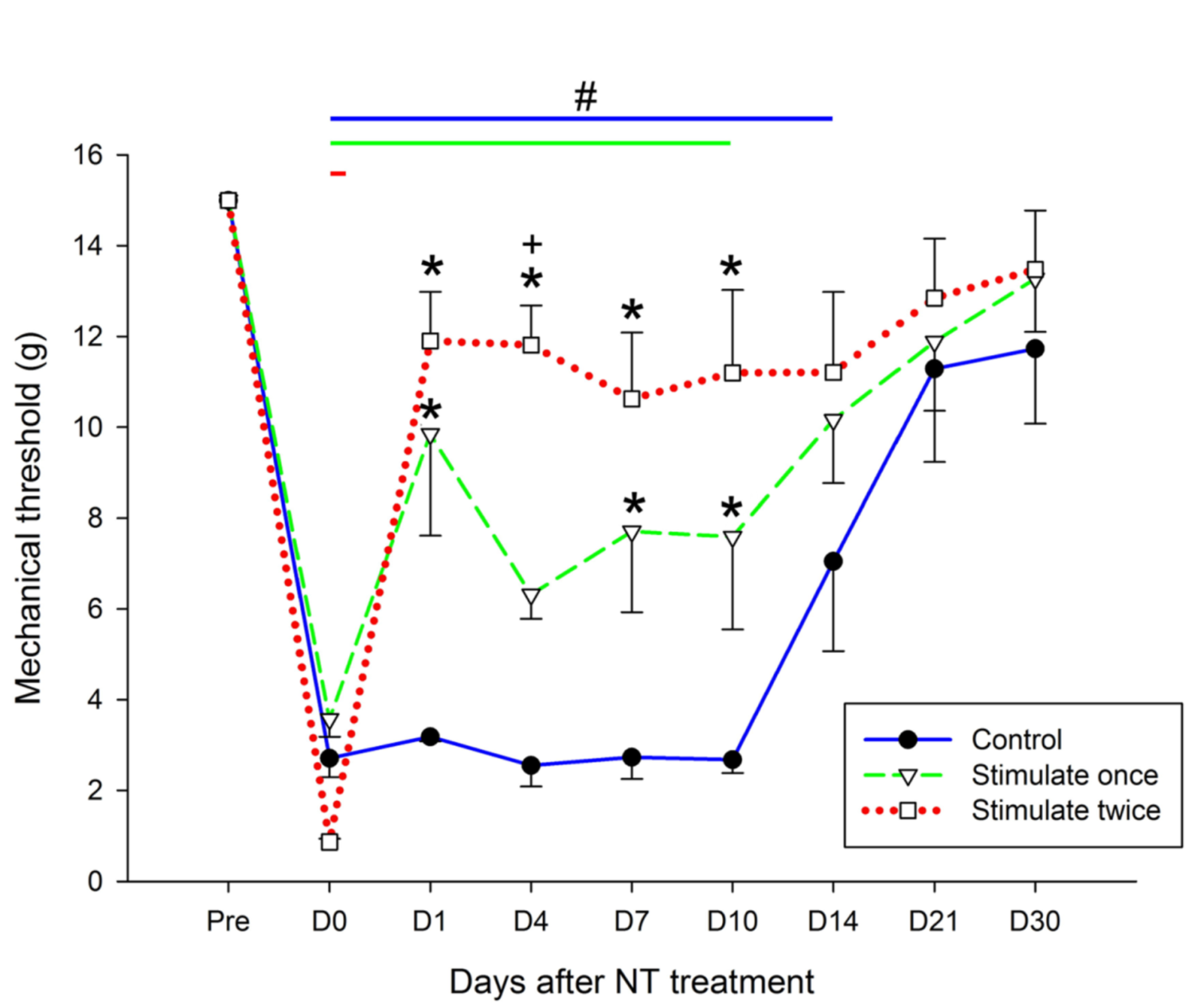
**

NT-like therapy reversed mechanical sensitization of the hind paw, which was produced via repeated injection of acidic (pH=4) solution into the gastrocnemius muscle of the rat. On the third day after the second acidic solution injection and the stable development of mechanical sensitization (as indicated by the decrease in the threshold force that produced hind paw withdrawal from 15 g to less than 4 g on Day 0), NT-like treatment was administered to the afflicted muscle at twofold the threshold intensity that caused gastrocnemius contraction (2T). Three groups of rats (2T twice, 2T once, and sham control; 6 rats each) were tested. The treatment day was denoted as Day 0. Note that two NP treatments (2T twice, the red line) reversed mechanical sensitization at D1, D4, D7 and D10.

*: Significantly different outcome between NT (p<0.01) and sham treatment on the corresponding day.

+: Significantly different outcome between NT (p<0.01) and 2T once.

#: Significantly different outcome between NT (p<0.01) and before acid injection (pre).

Two-way repeated-measures ANOVA (one-factor repetition) followed by multiple pairwise comparisons (the Tukey test) were used.

**Supplementary Text 1. Comparison of Studies One and Two**

The patients had a higher severity of pain and a longer chronicity on average in Study One than in Study Two. In Study One, nearly all control (PT-TENS) patients finished the allocated treatment; the NT (test arm) patients often underwent fewer sessions than the protocol allocation due to early successful completion, which erred on the conservative side in this superiority trial. In Study Two, most patients in both arms underwent the entire treatment course. The benefit of NT in Study One persisted at follow-up even though the average wait period of the NT patients before follow-up was longer than that of the PT-TENS patients (7 versus 5 weeks after the last session).

All PT patients in Study One remained on-protocol before they crossed over to NT after the 4-week washout period. Additionally, 72% (13 out of 18) of the PT patients in Study Two reported receiving off-protocol treatment between the first and second follow-ups due to their on-going pain: 6 patients received 6 or more additional PT sessions, 6 took analgesics or muscle relaxants, and 5 sought alternative treatments such as massage. These behavioural differences may have arisen for multiple reasons. (1) The accessibility of care: Taiwan offered full public health plan coverage, whereas health plans in California often included high deductibles. (2) ROM was measured in every session of Study One but not in Study Two; therefore, the NT therapists in Study Two might not have paid attention to the impaired muscles that caused the functional deficit until follow-up, reducing the functional improvement attained in Study Two. (3) The newly trained NT therapists may not have fully educated the patients about the importance of not stressing the treated area during the resting period, which may have led to the increase in repeated treatment sessions observed in Study Two. The differences in background characteristics may also explain the minor difference in outcomes between the two studies. However, the different actions taken by NT and PT-TENS patients in both studies corroborated

the difference in efficacy between the two treatments.

As all NT patients reported being nearly or completely pain-free in Study One, the NT patients were less motivated to return for follow-up (an average of 11 weeks after baseline or 7 weeks after the last NT treatment) than the PT-TENS patients (an average of 4 weeks after the last PT-TENS treatment).

Note that the difference in exposure to treatment between the control (PT-TENS) and test (NT) subjects erred on the conservative side of this superiority trial.

**Supplementary Text 2. Additional Results from Studies One and Two**

Six patients in Study One reported both neck pain and shoulder pain, although none of these patients met the severity threshold in the selection criteria for both areas, so none were registered as suffering from both conditions. The patients in the NT arm and the PT-TENS arm reported similar BPI “at its worst” scores (7.7 vs. 8.1) and QOL (26.4 vs. 27.5) at baseline. Note that the patients in the test (NT) arm appeared to have worse functional impairment at baseline (10.2) than those in the control arm (PT-TENS, 5.8) because the patients were randomized only according to the primary measure (BPI “at its worst”). The incidental difference was not statistically significant and erred on the conservative side. On average, the patients in the NT and crossover-to-NT arms of Study One received fewer treatment sessions (2.3 and 2.1 sessions, respectively) than those in the PT-TENS arm (2.9) due to early successful completion, and this result erred on the conservative side in this superiority trial.

NT reduced the pain score to zero within a relatively short time. In Study One, 21%, 53% and 74% of the NT patients became pain-free (all BPI measures=0) within one, two, or three treatment sessions, respectively, and these effects were persistent, as shown in Supplementary Figure 1. The remaining patients were nearly pain-free (BPI ≤2) after the third NT session. NT was completed in 2.26 sessions on average. No PT-TENS patients reported early successful completion. Relapses of pain in the NT arm occurred in three cases after the first or the second session (Supplementary Figure 1) associated with whiplash in a car accident, noncompliance with instructions and newly surfaced pain as reported by the patients. Incremental changes in BPI “right now” in selected patients in the NT arm (Supplementary Figure 2) revealed typical patient experiences before and after each session.

**Supplementary Text 3. Effects of NT versus Sham Therapy on Mechanical Hyperalgesia in Rats**

*Methods*

A randomized controlled experiment examining an NT-like procedure compared to sham control treatment was performed on adult male Sprague-Dawley rats, which were purchased from BioLasco Company in Taiwan. Groups of 2 to 3 rats were housed together in plastic cages and were placed in a temperature- and humidity-controlled room (23 ± 2°C and 55 ± 5%, respectively) with a 12-h light/dark cycle (lights on at 06:00 h). Food and water were available *ad libitum*. All animal care and experimental procedures were approved by the Institutional Animal Care and Use Committee of National Taiwan University (Approval No. NTU-103-EL-69).

The rats weighed between 260 and 400 g at the beginning of the experiment. The repeated acid injection-induced hyperalgesia model was used to induce chronic mechanical hyperalgesia according to the Sluka model^6,7,49^. Acidic (pH 4.0) saline was prepared by adding HCl droplets to sterile saline. Two dosages of 100 μL of this acidic saline solution were injected into the middle of the left gastrocnemius muscle of the rat under 4% isoflurane anaesthesia. The two intramuscular injections were separated by an interval of 5 days. Mechanical sensitivity was assessed based on the threshold force eliciting a lift of the hind limb via stimulation of the heel region of the left hind paw with von Frey filaments (North Coast Medical, Inc., Morgan Hill, CA, USA). Each rat was individually placed on an elevated wire mesh floor in a transparent acrylic box (dimensions of 21 cm × 12 cm × 14 cm) and were allowed to acclimate; each rat withstood 15 g filament stimulation before the acid injections. On each test day, the rat was placed in the same chamber to acclimate for 10 min. We applied von Frey filaments of various bending forces (0.6, 1, 2, 4, 6, 8, and 15 g). Brisk withdrawal or paw flinching was regarded as a positive response. The threshold force was determined using the up-down procedure described by Chaplan et al.^50^.

NT-like treatment was initiated on the third day after the second acid injection. The Noxipoints of rats has the same definition as in human patients, i.e., they approximated the locations of the origin and the insertion of a muscle group, coinciding with the sites containing high concentration of nociceptors in the muscle^30^. In the repeated acid injection model of rat muscle hyperalgesia, the gastrocnemius-soleus muscle (GS) is injured. The Noxipoints of the GS are: (1) the junction between calcaneal tendon and the GS proper (i.e., the insertion of GS), and (2) the attachments of the lateral and the medial heads of GS (i.e., the origin of GS). Under 4% isoflurane anaesthesia, a smaller cylinder-shaped piece of aluminum foil was placed around the insertion of GS, and the second wider piece of aluminum foil surrounding the origin of GS as the pair of Noxipoints for the two poles of our electrical stimulation. The arrangement of electrical stimulation of the paired Noxipoints of the injured muscle from the two ends is optimal for activating muscle nociceptors. Both pieces were half-circle shaped, and care was taken to secure contact of the half circle with the portion of the leg corresponding to GS. Electrical stimulation was applied for three minutes. The threshold intensity that elicited ipsilateral lower leg contraction was defined as 1T, and an intensity (measured using an oscilloscope) of 2T was applied to approximate the NT-like treatment, based on the relative intensities of NT stimulation observed in human patients (Figure 4). One of the two test groups was treated with NT once on Day 0, and the other group was treated with NT twice, once each on Day 0 and Day 3. The sham group underwent anaesthesia and electrode placement without stimulation. The mechanical threshold of the hind paws to von Frey stimulation was measured at 1, 4, 7, 10, 14, and 21 days after NT-like treatment. The period of observation of induced hyperalgesia (Days 0-10) was dictated by the time point at which the control group exhibited naturally reduced sensitivity (after Day 10). Two-way repeated-measures ANOVA (one-factor repetition) followed by multiple pairwise comparisons (the Tukey Test) were used to analyse the data.

*Results*

NT-like therapy significantly reversed mechanical sensitization of the rat hind paw (80.7% reduction in the 2T-twice group at Day 3, persisting through Day 30; p<0.01; see Supplementary Figure 3), but sham therapy did not induce any change in mechanical sensitization.

**Supplementary Text 4. Statistical Analysis Plans (Prior to the Analyses)**

**Statistical Analysis Plan for Study One**

In this superiority trial, analysis of the post-treatment outcome measures will consider NT as the test arm. The PT-TENS treatment outcome measures will be considered as the primary control. The history of failed treatments prior to T0 in the NT group will be used as the secondary control.

A statistical comparison of outcome measures between NT (after treatment) and PT-TENS will be conducted. A statistical comparison of outcome measures before and after the test treatment will be conducted for the cases in the NT arm. The subjects will be stratified according to a BPI “at its worst” of 7.5 before randomization. An allocation ratio of 2:1 (NT:PT-TENS) will be used due to the substantial effect of NT in preliminary trials. Additionally, PT and TENS had been thoroughly studied in numerous randomized studies in the literature.

The primary analysis will compare all outcome measures at follow-up to those at baseline for each arm using paired t tests. Changes in these outcome measures between the NT arm and the PT-TENS arm during the first stage, between crossover-to-NT and PT-TENS patients, and between crossover-to-PT and NT patients during the second stage will be further compared using t tests. All comparisons will be tested at a two-sided significance level of 5%. The secondary analysis in Study One will further evaluate the incremental changes in these outcome measures during the treatment course. All estimated p-values will be two-tailed.

The effective sample size (before dropouts were considered) was 28. We planned to enrol approximately 35 (20-45) patients at a 2:1 randomization ratio of NT: PT. This sample size will provide 90% power to detect a 4-point mean difference between the two arms in the change in BPI “at its worst” from baseline at a two-sided significance level of 5%, assuming a standard deviation of the change from baseline in BPI “at its worst” in both arms of three points and a dropout rate of approximately 20%.

Statistical analysis will follow the last-observation-carried-forward (LOCF) method in the primary analysis. Patients not receiving any post-baseline observations will be excluded.

**Statistical Analysis Plan for Study Two**

NT and PT-TENS will be used as the test and control arms, respectively, in both the primary and secondary analyses in this superiority trial.

The effects of PT-TENS and NT will be compared based on two-sample hypothesis-testing methods. The level of significance was set at 0.05. All estimated p-values were two-tailed. Subjects not receiving any post-baseline observations will be excluded. Missing data will be imputed using the LOCF method.

A statistical comparison of the outcome measures between NT (after treatment) and PT-TENS will be conducted. A 1:1 randomization ratio will be used, as the contrast between the two arms was assumed to be less than that in Study One (due to the lower effectiveness of NT performed by newly trained NT therapists and the less heavily treated patients with less severe pain at baseline).

The primary analysis will compare all outcome measures at follow-up to those at baseline for each arm using paired t tests. Changes in these outcome measures will be further compared using t tests between the NT and PT-TENS arms. All comparisons will be tested at a two-sided significance level of 5%.

The effective sample size (before dropouts were considered) was 44. The plan was to enrol up to 80 patients (at a 1:1 randomization ratio of NT:PT). This sample size was expected to provide 90% power to detect a 3-point mean difference between the two arms in the change in BPI “at its worst” from baseline at a two-sided significance level of 5%, assuming a standard deviation of the change from baseline in BPI “at-its-worst” in both arms of 3 points and a conservative dropout rate of approximately 40%.

The enrolment size of Study Two was larger than Study One because (1) the assumed difference of the effect size in the two arms was expected smaller in Study Two due to the enrolment of less-heavily pretreated patients (who may also respond to PT) and expected less effectiveness of the newly trained NT therapist; and (2) a higher dropout was anticipated due to the high (and free) accessibility of multiple treatment options of health care system in Taiwan.

Statistical analysis will follow last-observation-carried-forward for both NT and PT-TENS arms. Patients without any post-baseline observation will be excluded.

**Final Statistical Analysis Plan and Changes in Both Studies One and Two**

In Study One, two QOL measures in addition to BPI “interference with functions” (i.e., the Neck Disability Index (NDI) and the Shoulder Pain and Disability Index (SPADI)) were assessed, and data were collected. However, the initial analyses of the NDI and the SPADI revealed changes similar to those observed for BPI “interference with function” and did not provide additional insight. Thus, these results were not included in this report.

Missing data were imputed using the LOCF method. Patients not receiving any post-baseline assessments were excluded. Patients with early successful completion (i.e., no further treatment sessions were needed) reported their pain level and QOL by phone; the ROM measures had to be assessed in person and were thus missing for these sessions. These missing interim ROM measures were imputed backward based on the measures observed at follow-up (presumably the worst observation). Such imputed data were used only in the secondary analysis (i.e., the incremental analysis) of Study One.

Due to the relatively small sample size of the PT-TENS group in Study One, we conducted the Wilcoxon signed-rank test in addition to the one-sample paired t test for the primary analysis to compare the outcome measures at follow-up to those at baseline for each arm and at each stage. The changes in these outcomes were further compared between the NT arm and the PT-TENS arm during the first stage and between the crossover-to-NT and PT-TENS patients during the second stage using two-sample t tests and Wilcoxon rank-sum tests. The p-values from both the t test and the Wilcoxon ranked-sum test fell in the same range (as described in Tables 2 and 3). Only t tests were conducted in Study Two, as the sample distribution clearly passed the normality test.

As ROM was assessed in every intermediate session of Study One but only in the first session of Study Two, patients not receiving any ROM observations during the follow-up of Study Two were excluded from the ROM analysis (but may have been included in the BPI analyses). In Study Two, all 20 NT and 18 PT patients were included in the BPI analyses. Four NT patients and one PT patient who did not return for Follow-up 1 or Follow-up 2 were excluded from functional-impairment (ROM) analysis. The protocol required the collection of the BPI data before each session and at each follow-up but required the collection of ROM data only at baseline and at each follow-up. As these five patients did not return for either follow-up, no ROM measure aside from the pre-treatment assessment was available. Imputing data from the pre-treatment baseline for the missing data at Follow-up 1 and Follow-up 2 would not be appropriate, as their corresponding BPI scores showed a substantial pain reduction.

Note that the actual dropout rate (15%) in Study Two was much lower than the anticipated rate (40%+) in the original plan.

Study One was terminated when the patient count for the NT arm in the protocol (N for NT=25) was reached. Study Two was terminated when the IRB-approved end-date of the protocol was reached.
